# Supplementary material for: Chrysoprase color grading with machine learning: A systematic approach
Source: PLoS One. 2026 May 15;21(5):e0349205. doi: 10.1371/journal.pone.0349205 (PMC13178869; doi:10.1371/journal.pone.0349205)
Supplement: S2 Table — (DOCX) [file pone.0349205.s002.docx]

Table S2. Hyperparameter search space used in Bayesian optimization for the five machine learning algorithms.

| **Algorithm** | **Hyperparameter** | **Search Range / Options** |
| --- | --- | --- |
| SVM | Kernel function | Gaussian, Linear, Quadratic, Cubic |
|  | Box constraint level | 0.001 – 1000 |
|  | Kernel scale | Auto, Manual |
|  | Multiclass coding | One-vs-one, One-vs-all |
|  | Standardize data | Yes / No |
| KNN | Number of neighbors | 1 – Infinity |
|  | Distance metric | Euclidean, Cityblock, Chebyshev, Minkowski (cubic), Mahalanobis, Cosine, Correlation, Spearman, Hamming, Jaccard |
|  | Distance weighting | Equal, Inverse, Inverse squared |
|  | Standardize data | Yes / No |
| Random Forest | Ensemble method | Bag, AdaBoost, RUSBoost |
|  | Maximum splits | 1 – Infinity |
|  | Number of trees | 1 –Infinity |
|  | Number of variables sampled per split (mtry) | 0.01 – 1 |
| ANN | Number of fully connected layers | 1 – 3 |
|  | Hidden layer size (each layer) | 1 –Infinity |
|  | Activation function | ReLU, Tanh, None, Sigmoid |
|  | Maximum iterations | 1000 |
|  | Regularization strength (Lambda) | 0 – Infinity |
|  | Standardization | Yes / No |
| Logistic Regression | Solver | SGD, LBFGS, SpaRSA |
|  | Regularization | L2 (ridge), L1 (lasso) |
|  | Regularization strength (Lambda) | 0 –Infinity |
|  | Beta tolerance | 0.0001 |
|  | Multiclass coding | One-vs-one, One-vs-all |
