# Supplementary material for: Chrysoprase color grading with machine learning: A systematic approach
Source: PLoS One. 2026 May 15;21(5):e0349205. doi: 10.1371/journal.pone.0349205 (PMC13178869; doi:10.1371/journal.pone.0349205)
Supplement: Attachment — Submitted filename: REVIEWER.docx [file pone.0349205.s004.docx]

Dear Editor

While reviewing the article titled "Chrysoprase color grading with machine learning: a systematic approach" that I am refereeing, I noticed that especially the conclusion section was written by artificial intelligence. I am presenting you with the screenshots as evidence by attaching them to the Word file.

It is unacceptable that the conclusion, which is one of the main sections of an article, was produced by artificial intelligence in this way. This is an approach that goes against the essence of scientific writing

For this reason, I think that the article in question is not worth reviewing and should not be published.
