# Supplementary material for: Chrysoprase color grading with machine learning: A systematic approach
Source: PLoS One. 2026 May 15;21(5):e0349205. doi: 10.1371/journal.pone.0349205 (PMC13178869; doi:10.1371/journal.pone.0349205)
Supplement: Attachment — Submitted filename: response to reviewers.docx [file pone.0349205.s006.docx]

Dear Dr. Konys,

Thank you very much for the opportunity to revise our manuscript. We appreciate the constructive comments and suggestions provided by you and the reviewers.

We will carefully address all the points raised during the review process and revise the manuscript accordingly. The revised manuscript and the detailed response to the reviewers will be submitted through the system within the requested timeframe.

Thank you again for your consideration of our work.

Kind regards,
Yuansheng Jiang

Reviewer #1

I have encountered some negative comments regarding your article. I do not think it is possible to publish your article in its current form. Please review the comments carefully.

**Response:** We respectfully clarify that the scientific content of this manuscript, including the experimental design, data collection, data analysis, and interpretation of the results, was entirely developed and written by the authors based on their original research. The study is grounded in experimental measurements of chrysoprase samples and independent data analysis conducted by the research team.

The manuscript was initially drafted by the authors in their native language. To improve the clarity and readability of the English text, AI-assisted tools were used only for translation and language polishing. These tools were applied solely to refine grammar and wording. They were not used to generate scientific ideas, design experiments, analyze data, or produce the scientific conclusions presented in the manuscript.

The authors fully understand the importance of academic integrity in scientific publishing and take complete responsibility for the originality, accuracy, and authenticity of the work reported in this manuscript.

Reviewer #2:

1. Broaden Sample Representation for Enhanced Generalizability
The study relies on 51 chrysoprase samples exclusively sourced from Australia, which limits the model’s ability to account for color variations across global deposits. Chrysoprase from regions like Tanzania, Poland, or Kazakhstan often exhibits distinct color traits due to differing geological conditions and nickel content—factors that directly influence its market valuation. Expanding the sample set to include these international sources would ensure the grading system reflects real-world diversity. Additionally, focusing solely on cabochon-cut specimens with uniform color overlooks common natural variations like subtle color zoning or minor inclusions. Incorporating such samples would test the model’s robustness in practical, non-ideal scenarios, making it more reliable for industry use.

**Response:** We sincerely thank the reviewer for this valuable suggestion regarding the representativeness of the chrysoprase dataset and the applicability of the proposed grading model to natural gemstones.

(1) Geographic origin of samples

We agree that chrysoprase from different deposits (e.g., Tanzania, Poland, and Kazakhstan) may show variations in color characteristics due to differences in geological environments and nickel distribution. Expanding the dataset to include a broader range of geographic sources would indeed be beneficial for evaluating the global generalizability of the proposed grading framework.

However, it is important to note that reliable geographic origin determination for chrysoprase remains challenging. Previous mineralogical and isotopic studies have attempted to distinguish chrysoprase from different deposits, but these investigations generally report no clear isotopic signatures that allow consistent discrimination between geographic sources [1]. As a result, chrysoprase origin determination is currently not routinely achievable in gemological practice.

In this study, Australian chrysoprase was selected primarily because it represents one of the most widely studied and commercially important sources and exhibits the characteristic apple-green color range associated with chrysoprase. The primary goal of this work is therefore to establish a methodological framework for machine-learning-based chrysoprase color grading, rather than to construct a geographically comprehensive grading standard. We agree that future work should incorporate samples from additional deposits to further evaluate the robustness of the model.

(2) Use of uniformly colored cabochon samples

We also appreciate the reviewer’s comment regarding the use of uniformly colored cabochon specimens. In the present study, samples with relatively uniform color were intentionally selected due to limitations of the spectrophotometric measurement method. Color measurements were conducted using an X-Rite SP62 spectrophotometer with a 5 mm measurement aperture, which records reflectance spectra from a single measurement area. When strong color zoning or heterogeneous inclusions occur within the measurement spot, the recorded spectrum represents a spatial average of multiple color regions and may not accurately represent any specific perceived color of the gemstone.

To ensure that the measured CIELAB values correspond reliably to the perceived color of the specimens, relatively homogeneous cabochon samples were therefore used in this initial study. Establishing the grading model under controlled color conditions allows the relationship between colorimetric parameters and classification results to be evaluated more clearly.

We agree that natural chrysoprase may exhibit color zoning or inclusions in practical gemological contexts. Incorporating such samples would be valuable for testing the robustness of the grading system under real-world conditions. Future research will therefore aim to include more heterogeneous specimens and explore multi-point spectrophotometric measurements or imaging-based color analysis, which can better capture spatial color variation. This limitation and the corresponding future research directions have also been clarified in the Conclusion section of the revised manuscript.

2. Strengthen Alignment Between Model Output and Industry Practice
While the logistic regression model delivers impressive technical performance, the manuscript does not clearly connect its color grading outcomes to standard gemological workflows. Gemologists and appraisers rely on intuitive, perceptually meaningful criteria—not just numerical Lab* values—to assess color quality. For example, explaining how the “Fancy Intense,” “Fancy,” and “Fancy Deep” categories map to existing trade terminology or price differentials would bridge this gap. Supplementing with a direct comparison between the model’s results and evaluations from certified gemologists would also validate its relevance. Without this real-world context, the tool risks being seen as academically sound but impractical for day-to-day gem grading.

**Response:** We thank the reviewer for this insightful comment regarding the connection between the proposed grading framework and practical gemological workflows.

At present, chrysoprase does not have a widely accepted standardized color grading system in gemological practice, and color evaluation is largely based on subjective visual judgment. One motivation of this study is therefore to explore whether colorimetric measurements combined with machine learning can provide a more objective framework for describing chrysoprase color variation. The CIELAB color space used in this study was specifically designed to approximate perceptual color differences and is widely used in color science to quantitatively represent human color perception.

To facilitate communication with gemological practice, the three clusters identified in this study were assigned descriptive names—“Fancy Intense,” “Fancy,” and “Fancy Deep”—referencing the widely used GIA terminology for colored diamonds. These terms are intended as descriptive labels for relative lightness–chroma differences rather than as a direct application of the diamond grading system.

Regarding the reviewer’s suggestion to relate the grading results to market price, chrysoprase currently lacks a transparent or standardized pricing system, as prices are typically determined through private trade and influenced by multiple factors beyond color.

The reviewer also suggested comparing the model results with evaluations from certified gemologists. While such comparisons would certainly be valuable, conducting a structured expert evaluation requires carefully controlled viewing conditions and dedicated perceptual experiments, which are beyond the scope of the present study. Nevertheless, the proposed framework provides a reproducible and objective method for describing chrysoprase color variation, which may serve as a useful reference for future development of practical grading systems. This aspect will be considered in future work.

3. Address Practical Accessibility of the Grading Tool
The proposed color grading app requires input of Lab* values measured with an X-Rite SP62 spectrophotometer—a specialized piece of equipment most jewelers or small-scale traders do not own. This limits the tool’s widespread adoption. To enhance usability, the authors should test the model’s performance with data from more accessible devices, such as portable colorimeters or calibrated smartphone cameras. Providing guidance on how to obtain accurate color data without high-end equipment would make the tool actionable for a broader audience. Additionally, including a user-friendly interface demonstration or step-by-step guide for non-technical users would lower barriers to adoption, ensuring the research translates into tangible industry value.

**Response:** We thank the reviewer for this valuable comment regarding the practical accessibility of the proposed color grading tool.

It should be clarified that the X-Rite SP62 spectrophotometer used in this study is a portable handheld instrument designed for field measurements, and is widely used in industrial color quality control and colorimetric research.

In gemological practice, objective color grading and certification are typically performed by professional gemological laboratories or third-party certification institutions, where calibrated instruments and standardized procedures are used to ensure reliable and reproducible results. In this context, the use of a spectrophotometer for colorimetric measurement is consistent with current professional practices for gemstone evaluation.

We nevertheless agree that exploring alternative approaches for obtaining colorimetric data from consumer imaging devices, such as calibrated smartphone cameras, could further expand the accessibility of the proposed framework. However, achieving reliable colorimetric accuracy with such devices remains challenging due to variations in illumination conditions, camera sensors, and calibration procedures. This represents an interesting direction for future research.

Regarding usability, the proposed grading tool has been implemented as a publicly accessible application, where users only need to input the measured L^*^, a^*^, and b^*^ values to obtain the corresponding color category, keeping the workflow straightforward once colorimetric data are available.

4. Relevant References to Strengthen Methodological Context
To better contextualize the study’s core contribution—applying a systematic, data-driven classification framework to solve a longstanding industry challenge of subjectivity—it is recommended to cite the following two articles in the discussion section. These references align with the study’s emphasis on rigorous, cross-disciplinary methodology and standardization:
A comprehensive review on targeting diverse immune cells for anticancer therapy: Beyond immune checkpoint inhibitors（Crit Rev Oncol Hematol. 2025）This review advocates for moving beyond single-target approaches to develop multi-faceted, standardized strategies for complex biological classification—an idea directly paralleled in this study. The authors of the anticancer review highlight that relying on isolated markers (analogous to traditional subjective gem color assessment) leads to inconsistent outcomes, while integrating multiple data streams and systematic validation (like the combination of K-means clustering, Fisher discriminant analysis, and Bayesian optimization here) enhances reliability. Citing this work reinforces the broader significance of the current study’s methodology: just as the anticancer field benefits from standardized, multi-dimensional classification, gemology gains from objective, data-integrated grading that reduces human bias. This cross-disciplinary connection underscores the generalizability of systematic classification frameworks across fields with subjective evaluation challenges.
The role of cisplatin in modulating the tumor immune microenvironment and its combination therapy strategies: a new approach to enhance anti-tumor efficacy（Ann Med. 2025）This article focuses on optimizing therapeutic outcomes through strategic method combination and validation—an objective shared by the current chrysoprase grading study. The anticancer research emphasizes that combining complementary tools (e.g., drug therapy with immune modulation) addresses limitations of single-method approaches, much like how this study integrates colorimetric data, unsupervised clustering, and supervised machine learning to overcome the flaws of manual color grading. Additionally, the article stresses the importance of translating technical advancements into practical, standardized tools—mirroring this study’s development of a publicly accessible grading app. Citing this reference strengthens the discussion of the current work’s impact: both studies demonstrate how rigorous method integration and practical tool development can transform fields reliant on inconsistent, subjective assessments, making the gemology framework more relatable to broader scientific discourse on standardization.

**Response:** We thank the reviewer for the suggestion to include additional references to strengthen the methodological context of this study.

The recommended articles focus on anticancer therapy and tumor immunology, which belong to a substantially different research field from the present work in gemology and colorimetric classification. While we appreciate the reviewer’s intention to highlight potential cross-disciplinary methodological parallels, the specific scientific contexts and research objectives of these studies differ considerably from the scope of the current manuscript.

Therefore, these references were not directly incorporated into the manuscript. Instead, we have further clarified the methodological framework of the present study and its contribution to objective and data-driven color classification in gemology in the discussion section.

Reviewer #3:

1. The training set is composed entirely of 676 synthetic GemDialogue-derived points while the test set is only the 51 real chrysoprase samples. This design risks domain mismatch (synthetic vs real) and over-optimistic generalization claims. The perfect classification of the 51 samples by the final LR model is surprising and may reflect leakage, overly narrow class boundaries, or lack of realistic variance in the training set. Please (a) clarify the rationale for treating all real samples as a pure test set, and (b) repeat model evaluation using cross-validation schemes that incorporate the real samples into training folds (for example: stratified k-fold where both synthetic and real samples are mixed during fold assignment, or leave-one-out on real samples). Report results of these additional evaluations and discuss differences.

**Response:** We sincerely thank the reviewer for raising this important concern regarding the potential domain mismatch between synthetic and real samples.

(a) Rationale for using real samples as a pure test set

The primary objective of this study is to develop a color grading model that can generalize to unseen chrysoprase samples. The 676 GemDialogue-derived color points were designed to systematically simulate the perceptual green color space relevant to chrysoprase, thereby forming a comprehensive calibration domain. The 51 natural chrysoprase samples were therefore reserved as an external test set to evaluate the model’s performance on real-world data.

This design intentionally follows a calibration–validation framework, where synthetic color references are used to define grading boundaries, while natural samples serve as an independent validation set to assess generalization performance.

(b) Additional cross-validation incorporating real samples

To directly address the reviewer’s concern regarding potential domain mismatch and overly optimistic performance, we conducted an additional stratified five-fold cross-validation on the combined dataset (676 synthetic + 51 real samples; n = 727). In this evaluation scheme, both synthetic and real samples were randomly mixed during fold assignment while preserving class proportions. Each fold contained approximately ten real chrysoprase samples.

Using logistic regression with L2 regularization, the model achieved:

• Validation accuracy: 99.59%

• Macro F1-score: 99.59%

The confusion matrix showed only three misclassifications across all folds.

These results indicate that the model performance remains highly stable when real samples are incorporated into the training folds. The nearly identical performance compared with the original design suggests that:

1. The synthetic GemDialogue-derived color points provide a reasonable approximation of the real chrysoprase color distribution.
2. The perfect classification observed in the external test set is unlikely to be caused by data leakage.
3. The classification boundaries reflect the strong separability of the three chrysoprase color clusters in the CIELAB space, rather than artificially narrow decision boundaries.

These additional validation results have been incorporated into the revised manuscript (see the Selection and construction of machine learning models section).

2. procedure to create 2,730 overlay colors and then filter to 676 representative points requires more quantitative justification. Describe precisely (and provide code snippets or parameters) how the filtering used the L*, C*, h° bounds of the 51 samples. Discuss whether GemDialogue overlays realistically simulate cabochon optics (translucency, surface reflection, light scattering). If possible, validate the synthetic set by comparing distributions (e.g., kernel density or convex hull) of L*a*b* values between synthetic and measured samples. If synthetic data do not adequately cover real-world variability, consider augmenting the training set with measured variations (different orientations, multiple measurement locations per cabochon, different polish conditions, inclusion of proximate non-ideal samples).

**Response:** We sincerely thank the reviewer for this important and technically detailed comment. We have revised the manuscript to provide a fully reproducible description of the synthetic dataset construction and additional quantitative validation of its representativeness.

A total of 2,730 CIELAB color points were generated via systematic GemDialogue overlay combinations. To ensure that the synthetic dataset reflects the empirically observed chrysoprase color domain, we applied a deterministic filtering rule derived directly from the 51 measured samples.

For each measured sample, chroma and hue angle were computed as follows:

$$C^{*}=\sqrt{a^{*2}+b^{*2}}$$

$$h^{\circ}=arctan\frac{b^{*}}{a^{*}}$$

where the hue angle was computed using the four-quadrant inverse tangent and wrapped to 0°–360°.

The empirical bounds obtained from the 51 chrysoprase samples were:

L*: 38.50 – 76.14

C*: 18.20 – 46.96

h°: 143.22° – 178.40°

A synthetic point was retained only if it simultaneously satisfied all three constraints:

$$L^{*}\in\left[ L_{min}^{*},L_{max}^{*} \right] \wedge C^{*}\in\left[ C_{min}^{*},C_{max}^{*} \right] \wedge h^{^{\circ}}\in\left[ h_{min}^{\circ},h_{max}^{\circ} \right]$$

This strict AND-condition filtering reduced the dataset from 2,730 to 676 representative points. These explicit parameter values and filtering criteria have now been clearly described in the revised manuscript to ensure full reproducibility of the procedure.

Importantly, this procedure prevents the synthetic dataset from expanding beyond the empirical color support region defined by the measured chrysoprase samples. In this sense, the synthetic data densify the observed color region rather than extrapolating beyond it.

We acknowledge that GemDialogue overlays simulate color through planar transmissive stacking and therefore cannot fully reproduce the complex optical behaviour of polished cabochons, including subsurface scattering in microcrystalline quartz, specular surface reflection, thickness-dependent light transport, and inclusion-related heterogeneity. However, the objective of the overlay system in this study is not to model gemstone optics at the physical level, but rather to densely sample the perceptually relevant region of CIELAB color space associated with chrysoprase coloration for data-driven classification.

To directly assess perceptual coverage, we performed a nearest-neighbour analysis using the CIEDE2000 color-difference formula. For each measured chrysoprase sample (n = 51), the minimum ΔE_00_ relative to the synthetic dataset (n = 676) was computed. Results show that 47.06% of measured samples have a synthetic counterpart within ΔE_00_ < 1, which approximately corresponds to the perceptibility threshold under standard viewing conditions [2, 3]. Furthermore, 88.24% of samples fall within ΔE_00_ < 2, a tolerance range commonly adopted in industrial color evaluation [4]. The median minimum ΔE_00_ was 1.06, with the 95th percentile at 2.26, indicating substantial perceptual coverage of the measured chrysoprase color domain. These results suggest that the synthetic sampling grid closely approximates the empirical distribution of chrysoprase colors in CIELAB space. These additional validation results have been incorporated into the revised manuscript (see the Validation of synthetic color coverage section).

In addition to this geometric and perceptual validation, we examined whether the inclusion of synthetic points affects classification stability. The independent external evaluation (100% accuracy on the 51 real samples) and the stability observed under mixed stratified five-fold cross-validation (Macro F1-score = 99.6%) provide supporting evidence that the synthetic dataset does not appear to substantially affect the learned class boundaries.

Taken together, the strict bound-based filtering, ΔE_00_ nearest-neighbour coverage, independent real-sample evaluation, and stability under mixed cross-validation provide converging evidence that the synthetic dataset provides good coverage of the empirical chrysoprase color domain for the purposes of the present classification task.

Importantly, the synthetic points do not introduce additional structures or artificial clusters into the dataset. Instead, they act as a dense sampling grid within the empirically observed color domain defined by the measured chrysoprase samples. Therefore, the role of the synthetic dataset is to improve the continuity of the color space representation rather than to alter the underlying class structure.

Furthermore, the clustering used for labeling was performed on the combined dataset including the real samples, which further reduces the possibility that synthetic points alone determine the classification boundaries.

3. n = 51 real samples is small for definitive claims. Provide confidence intervals or statistical tests on classifier performance (e.g., bootstrap on the 51 samples, or uncertainty estimates from repeated resampling). Report per-class sample counts for the real samples and indicate whether any class is under-represented; if so, show class-wise precision/recall on the test set. The manuscript currently reports only overall macro F1 and an assertion that all 51 were correctly assigned; show the confusion matrix and per-class metrics for the test set and provide explanation if classes have very small n.

**Response:** We thank the reviewer for highlighting the importance of reporting statistical uncertainty and class-wise evaluation given the relatively small number of real chrysoprase samples (n = 51). In response, we have revised the manuscript to include confidence intervals, per-class sample counts, and detailed performance metrics for the independent test set.

The independent external evaluation on the 51 real chrysoprase samples yielded 100% accuracy (51/51 correctly classified). To account for sampling uncertainty associated with the limited sample size, we computed the 95% Wilson score confidence interval for the binomial proportion. The resulting interval is:

Accuracy = 100%
95% Wilson CI: 93.0%–100%

This indicates that, although all test samples were correctly classified within this dataset, the true underlying classification accuracy consistent with this sample size may be lower than 100%.

The distribution of the 51 real chrysoprase samples across the three color classes is as follows:

• Fancy Intense: n = 11
• Fancy: n = 32
• Fancy Deep: n = 8

The corresponding confusion matrix for the real-sample test set is:

Per-class recall values are therefore 1.00 for all three classes. To reflect the statistical uncertainty due to limited sample size, we also report 95% Wilson confidence intervals for the per-class recall estimates:

• Fancy Intense (n = 11):
Recall = 1.00 (95% CI: 0.74–1.00)

• Fancy (n = 32):
Recall = 1.00 (95% CI: 0.89–1.00)

• Fancy Deep (n = 8):
Recall = 1.00 (95% CI: 0.68–1.00)

As noted by the reviewer, the Fancy Deep class contains relatively few real samples, resulting in a wider confidence interval and therefore greater statistical uncertainty. This limitation has now been clarified in the revised manuscript.

Taken together, although the point estimate of the test accuracy is 100%, the reported confidence intervals provide a more statistically rigorous interpretation of classifier performance. Additional validation on larger and more diverse real-world datasets would further strengthen the generalizability of the proposed grading framework.

4. The number of clusters (three) was chosen using silhouette applied to only the 51 real samples; then k-means with k=3 was applied to the combined dataset to label synthetic points. This approach is reasonable but may bias labeling toward the real-sample distribution. Make explicit the assumptions and potential limitations of this choice. Also, adopting GIA “Fancy” nomenclature (developed for diamonds) for chrysoprase requires caution — justify this transfer more explicitly (e.g., perceptual studies or prior literature applying similar labels to coloured gemstones).

**Response:** We thank the reviewer for this important methodological comment. The number of clusters was determined using silhouette analysis applied to the 51 real chrysoprase samples. This design choice intentionally anchors the cluster structure to the empirical color distribution of measured chrysoprase rather than to the uniformly constructed synthetic dataset. Because the 676 synthetic points were systematically generated to densely sample the perceptual CIELAB region, they do not exhibit intrinsic clustering tendencies. Applying silhouette analysis directly to the synthetic dataset would therefore favor mathematically compact partitions that may not correspond to perceptually meaningful chrysoprase categories.

We acknowledge that determining the cluster number solely from the real samples introduces an anchoring assumption: namely, that the 51 measured samples adequately represent the perceptual diversity of chrysoprase color. If certain color variations are under-represented in this dataset, the derived cluster structure may not fully capture the broader population distribution. This limitation has now been explicitly discussed in the revised manuscript in the section “Labeling of color data”.

Regarding the use of the GIA “Fancy” terminology, we emphasize that its adoption in this study is intended as a descriptive reference for lightness–chroma relationships rather than as a direct transfer of diamond grading standards. In gemstone color research, lightness–chroma-based descriptors are commonly used to communicate perceptual color differences across materials. In the present study, the terms “Fancy Intense”, “Fancy”, and “Fancy Deep” are used only to describe the relative lightness–chroma structure identified among chrysoprase clusters in CIELAB space. To clarify this point and avoid potential misunderstanding, we have revised the manuscript and added supporting references where similar lightness–chroma–based descriptive terminology has been applied in colored gemstone colour studies.

5. Provide exact measurement geometry (you state SCI and D65, 2° observer) but clarify: measurement aperture size, number and location of measurement points on each cabochon (top only or multiple locations?), orientation control, whether measurements were taken through a mounting (e.g., on white card) or handheld, and repeatability (report standard deviation of triplicate measurements). Also indicate whether measurements were made on faceted or cabochon surfaces (you state cabochons but shapes differ); polishing and curvature affect measured color — discuss.

**Response:** We thank the reviewer for this valuable suggestion. We have revised the “Color measurement” section to provide complete methodological details.

Color measurements were performed using an X-Rite SP62 spectrophotometer with d/8° geometry in SCI mode. The measurement aperture was 5 mm. All specimens were polished cabochons (not faceted stones). Measurements were taken on the domed top surface at the apex (single standardized location) to ensure comparable geometry across samples.

During measurement, samples were placed on a Munsell N9.5 neutral background. The instrument was positioned perpendicular to the surface in contact mode (handheld). Prior to measurement, calibration was conducted following the manufacturer’s procedure using the white calibration standard (white ceramic tile) and black trap.

For each specimen, three consecutive measurements were acquired at the same location and the mean value was recorded. The individual triplicate values were not archived separately; therefore, standard deviations cannot be retrospectively calculated. According to the manufacturer’s specification, the short-term repeatability of the instrument is typically within ΔE^*^ ≤ 0.04 under repeat measurement conditions, indicating high instrumental stability. This clarification has been added to the manuscript.

We acknowledge that surface curvature and polishing can influence reflectance measurements due to geometric effects. To minimize this influence, measurements were consistently taken at the apex of the cabochon surface, and SCI mode (specular component included) was used so that the specular reflection from the polished surface was included in the measurement. This approach reduces sensitivity to variations in surface gloss and improves the comparability of color measurements between samples. While minor geometric effects cannot be completely eliminated, measurement conditions were standardized across all samples to ensure internal consistency. This discussion has been added to the manuscript.

6. Hyperparameters table is useful, but please provide (in methods or supplementary) the optimization ranges used for Bayesian optimization, the Bayesian optimizer settings (acquisition function, iterations), and the random seed(s). Confirm whether reported results are deterministic across seeds. Provide code or a README in the GitHub repo explaining how to reproduce training and evaluation exactly (including MATLAB versions and toolboxes used).

**Response:** We thank the reviewer for this valuable suggestion. We have revised the “Hyperparameter Optimization” section to provide complete methodological details.

**Bayesian optimization settings**
Hyperparameter tuning was performed using MATLAB R2024a Classification Learner with the default Bayesian optimization configuration. The acquisition function was set to Expected Improvement per Second (plus), and the maximum number of objective evaluations (iterations) was set to 30. The optimization ranges for each algorithm are summarized in the Supplementary Material (Table S3).

**Random seed and determinism**
To ensure reproducibility, a fixed random seed (rng(123,'twister')) was used in all experiments. Under this deterministic configuration, the training, cross-validation, and evaluation procedures are fully reproducible. Consequently, the macro F1-scores differ slightly from those reported in the previous manuscript version where no fixed random seed was used. The updated values have been revised in the “Selection and Construction of Machine Learning Models” subsection.

**Code and reproducibility documentation**
To ensure full reproducibility, the GitHub repository has been updated to include the MATLAB scripts required to reproduce the training and evaluation procedures. The repository now contains:

1. MATLAB training scripts for the five machine learning models (logistic regression, SVM, KNN, random forest, and neural network).
2. The dataset used in this study.
3. A detailed README file describing the computational environment and reproduction steps.

The README specifies the MATLAB version used (MATLAB R2024a), the required toolboxes (Statistics and Machine Learning Toolbox and Deep Learning Toolbox), the fixed random seed (rng(123,'twister')), and step-by-step instructions for reproducing the cross-validation and evaluation results.

7. The manuscript shows validation confusion matrices (via CV) but does not present the confusion matrix for the independent 51-sample test set other than stating perfect assignment. Include: (a) full confusion matrix for the 51 samples, (b) per-class precision/recall/F1, (c) visualization of decision boundaries in L*a*b* (or PCA) with real samples highlighted, and (d) calibration plots or probability outputs (for logistic regression) to assess classifier confidence.

**Response:** Thank you for this helpful suggestion. We have now added the requested evaluation details for the independent 51-sample test set in the revised manuscript (Section Selection and construction of machine learning models).

1. A full confusion matrix for the 51 real chrysoprase samples has been added in the revised manuscript (Fig. 9b).
2. Per-class precision, recall, and F1-score have been reported in a new table (Table 4).
3. A visualization of the classifier decision boundaries in PCA-reduced space with real samples highlighted has also been included (Fig. 9a).
4. To assess classifier confidence, we extracted the posterior class probabilities from the logistic regression–based ECOC classifier for the independent test set. The predicted probabilities for the assigned classes ranged from **0.6626 to 1.0000** (mean = **0.9752**, median = **0.9988**), indicating generally high confidence in the model predictions.

These additions improve the completeness and transparency of the model evaluation.

**Reference**

1.	Skrzypek G, Sachanbinski M, Jedrysek MO, editors. Oxygen isotope evidence for low temperature formation of chrysoprase. Oxygen isotope evidence for low temperature formation of chrysoprase; 2003: Polskie Towarzystwo Mineralogiczne.

2.	Luo MR, Cui G, Rigg B. The development of the CIE 2000 colour‐difference formula: CIEDE2000. Color Research & Application: Endorsed by Inter‐Society Color Council, The Colour Group (Great Britain), Canadian Society for Color, Color Science Association of Japan, Dutch Society for the Study of Color, The Swedish Colour Centre Foundation, Colour Society of Australia, Centre Français de la Couleur. 2001;26(5):340-50.

3.	Sharma G, Wu W, Dalal EN. The CIEDE2000 color‐difference formula: Implementation notes, supplementary test data, and mathematical observations. Color Research & Application: Endorsed by Inter‐Society Color Council, The Colour Group (Great Britain), Canadian Society for Color, Color Science Association of Japan, Dutch Society for the Study of Color, The Swedish Colour Centre Foundation, Colour Society of Australia, Centre Français de la Couleur. 2005;30(1):21-30.

4.	Commission Internationale de lE. Improvement to Industrial Colour-Difference Evaluation. Vienna: CIE Central Bureau, 2001 CIE Publication No. 142:2001.
